# Harmful Effects and Potential Benefits of Anti-Tumor Necrosis Factor (TNF)-α on the Liver

**DOI:** 10.3390/ijms19082199

**Published:** 2018-07-27

**Authors:** Loris Riccardo Lopetuso, Giammarco Mocci, Manuela Marzo, Francesca D’Aversa, Gian Lodovico Rapaccini, Luisa Guidi, Alessandro Armuzzi, Antonio Gasbarrini, Alfredo Papa

**Affiliations:** 1 Internal Medicine and Gastroenterology Department, Fondazione Policlinico Universitario A. Gemelli IRCCS-Università Cattolica del Sacro Cuore, 00168 Roma, Italy; lopetusoloris@libero.it (L.R.L.); manuelamarzo@gmail.com (M.M.); francesca.dav@hotmail.it (F.D.); gianludovico.rapaccini@policlinicogemelli.it (G.L.R.); luisa.guidi@policlinicogemelli.it (L.G.); alearmuzzi@yahoo.com (A.A.); antonio.gasbarrini@unicatt.it (A.G.); 2 Gastroenterology Unit, Brotzu Hospital, 09121 Cagliari, Italy; giammarco.mocci@gmail.com

**Keywords:** infliximab, adalimumab, etanercept, certolizumab pegol, golimumab, hepatitis B virus, hepatitis C virus, drug-induced liver injury, non-alcoholic fatty liver disease, alcoholic hepatitis, autoimmune hepatitis

## Abstract

Anti-tumor necrosis factor (TNF)-α agents represent an effective treatment for chronic inflammatory diseases. However, some concerns about their potentially undesirable effects on liver function have been reported. On the other hand, evidence of their therapeutic effects on certain liver diseases is accumulating. Many data showed the safety of anti-TNF-α in patients with chronic hepatitis B and C and in liver transplanted patients even if a strict follow-up and prophylaxis are recommended in well-defined subgroups. On the other side, anti-TNF-α-induced liver injury is not a rare event. However, it is often reversible after anti-TNF-α withdrawal. Anti-TNF-α agents have been tested in advanced stages of severe alcoholic hepatitis and non-alcoholic fatty liver disease. Limited data on the efficacy of anti-TNF-α in patients with autoimmune hepatitis and primary biliary cholangitis are also available. In this review, we explored the hepatic safety concerns in patients receiving anti-TNF-α agents with and without pre-existent hepatic diseases. In addition, the available evidence on their potential benefits in the treatment of specific hepatic diseases is discussed.

## 1. Introduction

In the last 20 years, anti-tumor necrosis factor (TNF)-α agents have been increasingly used for the treatment of patients affected by inflammatory bowel disease (IBD), such as Crohn’s disease (CD) and ulcerative colitis (UC), as well as rheumatologic and dermatological disorders such as rheumatoid arthritis (RA), juvenile idiopathic arthritis (JIA), ankylosing spondylitis (AS), psoriatic arthritis (PsA) and psoriasis. Currently, five TNF-α antagonists have been licensed by international regulatory authorities for clinical use: infliximab (IFX), adalimumab (ADA), certolizumab pegol (CER), etanercept (ETA) and golimumab (GOL). IFX (Remicade™, Janssen Biotech, Inc.) is a chimeric monoclonal immunoglobulin (Ig) G1 antibody composed of 75% human sequence and 25% mouse sequence, given intravenously. It is approved for RA, CD, UC, AS, PsA and psoriasis. ADA (Humira™, AbbVie Inc.) is a recombinant, fully human IgG1 monoclonal antibody licensed subcutaneously for RA, CD, UC and psoriasis, whereas CER (Cimzia™, UCB, Inc), a pegylated, humanised Fab fragment of an anti-TNF-α monoclonal antibody given subcutaneously, is licensed for RA, SA, and PsA and for CD only in the United States. Notably, all these drugs are able to neutralise both forms of TNF-α, either by binding the trans-membrane form (m-TNF-α) and/or by blocking the soluble form (s-TNF-α) [1], but only IFX and ADA induce apoptosis in T cells and monocytes [1,2]. The fourth anti-TNF-α agent ETA is a recombinant, covalently bound dimer of soluble p75 TNF-receptor fused to the Fc portion IgG1, which binds to soluble TNF-α, but not the membrane-bound TNF-α [3]. It is approved for RA, JIA, AS, PsA, and psoriasis. Lastly, GOL (Simponi™, Janssen Biotech, Inc.) is a human IgG1κ monoclonal antibody produced by a murine hybridoma cell line with recombinant DNA technology. It is administered subcutaneously and is indicated for the treatment of AR, PsA, AS and UC. With the widespread use of these anti-TNF-α agents, some concerns have been raised about their potential consequences in important clinical settings, such as in patients with hepatitis B virus (HBV) or hepatitis C virus (HCV) chronic infections [4]. In addition, hepatic toxicity of anti-TNF-α agents has generated attention, and an increasing number of cases of liver injury, also life threatening, have been reported [5,6]. On the other hand, anti-TNF-α agents have also been used for treating the severe stages of hepatic diseases such as alcoholic hepatitis (AH) [7,8] and non-alcoholic fatty liver disease (NAFLD) [9]. Recently, anecdotal evidences of efficacy of anti-TNF-α agents as rescue therapy in difficult-to-treat autoimmune hepatitis (AIH) and primary biliary cholangitis (PBC) have been reported [10,11,12] (Table 1).

Thus, in this clinical review we explore the potential risks of hepatic injury induced by TNF-α antagonists, both in patients without pre-existent hepatic disorders and HBV or HCV chronic infection carriers; and the available evidence on the beneficial use of anti-TNF-α agents for the treatment of some liver diseases, such as AH or NAFLD.

## 2. Methods

A literature search was conducted using Pubmed, up until November 2017. Original articles and reviews were identified using the key words: “anti-TNF-α”, “infliximab”, “adalimumab”, “certolizumab pegol”, “etanercept” and “golimumab” matched with each of the following key words: “viral chronic hepatitis”, “liver transplantation”, “drug-induced liver injury”, “autoimmune hepatitis”, “non-alcoholic fatty liver disease”, “alcoholic hepatitis”, “primary biliary cholangitis”. Additional articles were identified through a review of the reference lists of selected pertinent articles.

## 3. Chronic Viral Hepatitis

TNF-α is a major proinflammatory cytokine that plays an important role in the integrated host defence system against infectious diseases. Given the role of TNF-α on both the innate and adaptive immunity, it is not surprising that the major toxicity concern with the anti-TNF-α agents is the risk of serious infection. Indeed, in vivo animal studies and post-marketing analysis of anti-TNF-α agents have shown that the blockage of TNF-α signalling pathway leads to an increased susceptibility to a range of pathogens [13], in particular those able to survive intracellularly [14]. However, despite the fact that experimental and clinical data have clearly demonstrated an augmented susceptibility to bacterial, fungal and mycobacterial infections, the influence of anti-TNF-α agents on viral infections has not been extensively investigated. In particular, relatively few data are available for long-term safety of TNF-α antagonists in patients with chronic HBV or HCV infections. Furthermore, while the exclusion of any bacterial infection and the screening for tuberculosis is mandatory before starting biologic therapy [15], definite guidelines for the management of HBV or HCV infection in patients that need TNF-α antagonists have been published only recently. In particular, the European Crohn’s and Colitis Organization (ECCO) has promoted a consensus meeting for the prevention, detection and management of opportunistic infections in patients with IBD, including HBV and HCV [16].

### 3.1. Hepatitis C

It is estimated that there are nearly 100 million people worldwide with serological evidence of current or past HCV infection [17]. A growing body of evidence proves that in chronic HCV infection, TNF-α plays a role in the inflammatory response to HCV, including the mediation of apoptosis, but it does not play a part in the control of HCV replication [18,19]. In addition, it has been shown that TNF-α polymorphisms might have no effect on susceptibility to HCV infection and virus clearance [20]. On the other hand, a direct correlation between TNF-α and serum alanine aminotransferase (ALT) levels was found in patients with chronic HCV infection [21]. TNF-α is also implicated in refractoriness to interferon (IFN) therapy in HCV patients [22,23]. However, considered the availability of new extremely effective anti-HCV therapies, this study is of historical interest only. Currently, the issue of major interest is the safety of anti-TNF-α treatments in patients with concomitant chronic HCV infection. Limited and scattered data are available on this topic since most of them come from case reports and case series. In 2011, Brunasso et al. published a systematic review that included 153 HCV patients treated with anti-TNF-α agents between 1999 and 2010, aiming to assess the safety of this class of drugs [24]. Among these patients, 91 had RA, 7 had PsA, 8 had psoriasis, 8 had both psoriasis and PsA, 6 had CD and 14 had other chronic inflammatory diseases [24]. The mean duration of anti-TNF-α treatment was 11.9 months. 110 were treated with ETA, 34 with IFX and only 9 with ADA [24]. Authors found only one confirmed case of worsening HCV liver disease among 110 patients treated with ETA (with hepatic improvement after ETA withdrawal) and one probable case in patients treated with IFX where hepatic injury was not confirmed by liver biopsy or withdrawal of anti-TNF-α agent and subsequent improvement of ALT and HCV-RNA levels [24]. Lastly, in 5 patients an increase of transaminases did not correspond to increased viremia and liver biopsy was not performed [24]. After this review, other studies including a small series of patients were published, confirming the previous findings on the acceptable safety profile of this class of drugs in case of concurrent HCV-infection [25,26,27,28,29,30].

However, a word of caution is required for a number of reasons. First, these studies did not include pre- and post-treatment hepatic biopsies to exactly assess any worsening of liver damage due to anti-TNF-α therapy. Second, a long-term follow-up is not yet available and controlled trials are lacking. In addition, a small study including 6 cases with actively replicating chronic HCV infection on anti-TNF-α treatment for an associated RA showed the appearance of cryoglobulinaemia in 2/6 patients [31]. Thus, the authors concluded that anti-TNF-α agents may favour the emergence of a mixed cryoglobulinemia. Nevertheless, since then there have been no similar reports to confirm this argument. In conclusion, the available evidence shows that the use of anti-TNF-α agents is safe in HCV patients although the long-term effects of these drugs on the natural history of HCV infection are not completely elucidated.

### 3.2. Hepatitis B

In contrast to HCV, experimental studies in vitro and in animal models have shown that TNF-α plays a pivotal role in clearing HBV from infected hepatocytes by inhibiting the replication and stimulation of the HBV-specific T cell response [32,33,34,35,36]. In addition, patients with IBD are historically considered at increased risk of HBV infection due to the frequent need for surgical procedures and blood transfusion. The first studies have reported a significantly higher prevalence of HBV infection in IBD patients than in general population [37,38], with a prevalence of anti-HBc in 10.9% of IBD patients, compared to 5.1% of controls (*p* = 0.02) [38]. In contrast, other more recent epidemiological studies carried out in Western countries have reported HBV exposure rates in IBD patients comparable to or even lower than control populations [39,40,41]. These changes in epidemiology probably reflect the implementation of safety measures for blood transfusions and the global spread of vaccination against HBV. Reactivation of HBV infection in patients receiving chemotherapy for lymphoma or other malignancies, with viral antigens expression increase and a consequent development of immune-mediated liver injury is a well-known and frequently reported complication when immune reconstitution occurs [42,43,44]. In this scenario, the use of anti-TNF-α agents in patients with chronic HBV infection may lead to enhanced viral replication, which is followed by the development of immune-mediated injury when the inhibitory effects of therapy disappears. Available literature data in this field are for the most part case-report or retrospective studies and only a limited number of prospective cohort studies. In detail, in 2011 a revision including overall 257 cases was published. Among these, 89 patients were HBsAg+ carriers and 168 anti-HBc+ subjects (resolved HBV infection, also defined as “occult carriers”) [45]. As expected, the majority of the reported cases of viral reactivation during anti-TNF-α therapy occurred in carriers of HBsAg (35/89, 39%), with the exception of few cases observed in patients with HBV occult infection (9/168, 5%) [45]. Acute liver failure was reported in 5 patients (4 died) in the group of HBsAg positive and in 1 patient among anti-HBc positive who died [45]. IFX was associated with a higher rate of induced liver disease compared with ETA, while no comparisons were possible with the other anti-TNF-α agents for the paucity of cases. Interestingly, despite the fact that HBV reactivation during therapeutic immunosuppression can be effectively prevented with the use of antivirals [46,47,48,49], among HBsAg positive patients the antiviral prophylaxis was administered in less than half of the cases (lamivudine in 35, entecavir in 3, and telbivudine in 1 case) [45]. In recent years, other studies were carried out for assessing the effect of anti-TNF-α therapy in patients with both HbsAg and anti-HBc positivity confirming the aforementioned findings [26,28,50,51]. Therefore, in the next paragraph we provide practical recommendations for the proper management of patients with positive markers of hepatitis B or C receiving anti-TNF-α agents as a treatment.

### 3.3. Recommendations for the Management of Patients with Hepatitis B Virus (HBV) or Hepatitis C Virus (HCV) Infection in Therapy with Anti-Tumor Necrosis Factor (Anti-TNF-*α*) Agents

**Figure 1:**
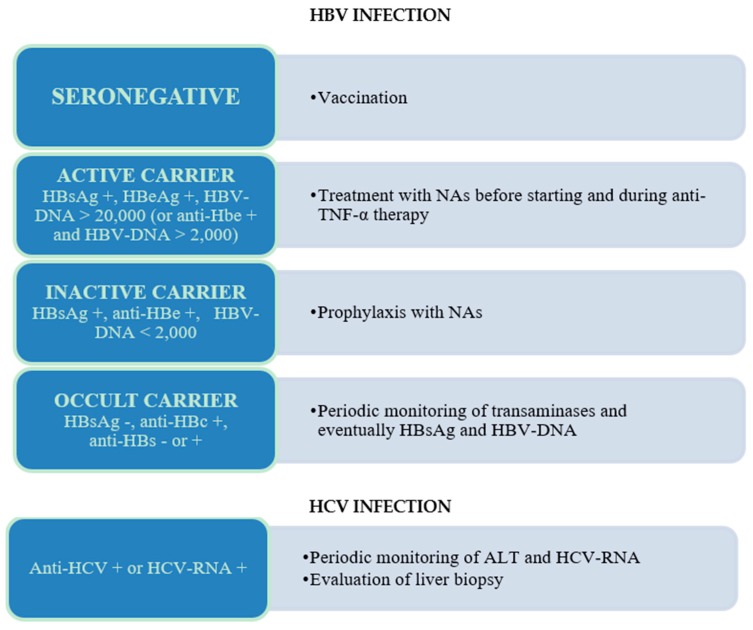
Recommendations for the management of patients according to the presence of different HBV and HCV markers. NAs, nucleoside analogues; ALT, alanine aminotransferase; TNF-α, tumor necrosis factor-α.

Currently, the presence of HBV or HCV infection should not preclude the therapy with an anti-TNF-α agent [16]. For HCV infection, there is no evidence that TNF-α blockers exacerbate the course of infection, even if the long-term effects are unknown. For this reason, in HCV-positive patients receiving long-term therapy with anti-TNF-α, periodic monitoring of ALT and HCV-RNA, and eventually follow-up liver biopsies are recommended, even if at present there is not a consensus for HCV screening before starting this treatment [16,52]. For HBV infection, because of the potential risk of reactivation related to steroids or immunosuppressant therapy, the ECCO guidelines recommend the assessment of HBV serological markers at IBD diagnosis and vaccination in all seronegative IBD patients [16]. In detail, the ECCO guidelines and the American Association for the Study of Liver Disease (AASLD) recommendations advise HBsAg, anti-HBsAg and anti-HBc testing for all patients before starting anti-TNF-α therapy [16,53]. Then, in HBsAg positive patients, HBV-DNA should be assessed to differentiate active carriers (HBV-DNA > 20,000 IU/mL in HBeAg positive patients, or >2000 IU/mL in anti-HBe negative patients) from inactive carriers [16,53]. Active carriers should be treated firstly for their active hepatitis with nucleos(t)ide analogues (NAs), such as lamivudine, entecavir or adefovir-pipivoxil, and then with anti-TNF-α for their underlining disease [54]. In HBV inactive carriers, it is recommended to begin the prophylaxis of viral reactivation with NAs prior to start the TNF-α antagonist (at least one week before) and to continue it for at least six to 12 months after the treatment is ended [53,54,55]. In the past, lamivudine was the most widely used NA for the prophylaxis of hepatitis B reactivation. However, its potent antiviral action is balanced by the concern of resistant mutants selection (50–60% at 4 years). For this reason, currently the use of NAs with low anti-viral resistance-rate, such as adefovir-pipivoxil or entecavir, is advisable. In occult HBV carriers (anti-HBc positive, HBsAg negative, anti-HBs positive or negative) on-going monitoring, with the early introduction of a NA in case of viral reactivation or re-appareance of HBsAg (seroconversion), seems to be the most cost-effective strategy [16,56]. For HBV vaccination, despite the aforementioned recommendations, the vaccine is still under-prescribed. In some European epidemiological studies, HBV vaccination rates in IBD patients ranges from 12% to 49%, depending on the mean age of population examined [39,40,41], suggesting that in these cohorts the majority of patients were vaccinated through mass vaccination programmes, rather than through a gastroenterologist intervention. On the other hand, the response rate to HBV vaccination in IBD patients seems to be quite low, mainly in those already receiving anti-TNF-α therapies, even when a double vaccine dose is administered [57,58,59,60] (Figure 1).

## 4. Liver-Transplanted Patients

Few data are available on the ideal management of IBD when combining anti-TNF-α therapies with immunosuppression after liver transplantation (LT). After LT, about 30% of patients report an improvement of IBD, while almost the same percentage of patients get worse. In addition, occurrence of de novo IBD can develop in approximately 14–30% of patients with primary sclerosing cholangitis (PSC) [61]. In this scenario, UC and CD may be refractory to standard treatment despite the use of post-LT immunosuppressive drugs and thus need the use of anti-TNF-α drugs.

A case series evaluated six patients with IBD who required LT for PSC (4/6), AIH (1/6) or biliary atresia (1/6) [62]. All of them were treated with IFX 5 mg/kg every 8 weeks with a standard induction regimen, with the exception of one patient. The duration of this therapy ranged from 8 weeks to 4 years with response rates similar to those of patients who had not previously been transplanted. In addition, post-transplant immunosuppressants, such as tacrolimus, mycophenolate mofetil (MMF), prednisone and cyclosporine, were safely administered to all subjects. In the follow-up period, one patient reported a systemic lupus erythematosus and another colorectal adenocarcinoma. Overall, in this study the combination of anti-TNF-α with standard immunosuppressants was thought to be safe and effective [62]. A retrospective study evaluated the efficacy and safety of anti-TNF-α therapy in 8 IBD patients who underwent to LT because of PSC (5/8) and concomitant cholangiocarcinoma (3/8). Clinical response was assessed in the 87.5% and mucosal healing in the 42.9%. 4 cases of infections were described in 3 patients (oral candidiasis, Clostridium difficile colitis, bacterial pneumonia and cryptosporidiosis). One patient had an Epstein–Barr virus positive lympho-proliferative disorder, while one death was observed because of complications from recurrent PSC [63]. Recently, a meta-analysis investigated the infection risk in 53 post-LT patients on anti-TNF-α medications compared with 23 post-LT subjects and 41 PSC-IBD not treated with anti-TNF-α. No significant difference for serious infections was assessed among groups [64]. Indeed, in the anti-TNF-α group the infection rate was 01.68 serious infections per patient year vs. 0.149 in the control patients (*p* = 0.886). After correction for time since transplant, in the anti-TNF-α group it resulted 0.194 vs. 0.115 in the non-exposed (*p* = 0.219) [64]. However, the small number of patients and the lack of randomized controlled trials included represent a limit and definitely require further larger well-designed studies.

Overall, anti-TNF-α therapy in post-LT IBD patients seems to be equally effective and safe despite the concomitant consumption of immunosuppressive medications. Nevertheless, caution should be used because of the risk of adverse effects, including cytopenia, opportunistic infections, and cancers [65].

## 5. Anti-TNF-α Liver Toxicity

Abnormalities in liver functions tests, including transient and self-limiting hypertransaminasemia, cholestatic disease and hepatitis can develop during treatment with anti-TNF-α and, in some cases, they could be severe and life threatening [6,7]. Indeed, for the first time in December 2004, a drug warning for IFX was issued by the Food and Drug Administration (FDA) following 35 voluntary post marketing reported events of severe hepatic reactions (plus 3 patients from controlled clinical trials) [66]. Since then, the FDA has reported more than 130 cases of liver injury resulting from either IFX or ETA treatment in post-marketing surveillance programs. Currently, all of the anti-TNF-α agents used in clinical practice have been associated with drug-induced liver injury (DILI). Mancini et al. analysed the main characteristics of IFX-related liver injury [67]. IFX can provoke both immuno-mediated and direct liver injury after a range of 1–12 infusions [67]. Although, either a hepatocellular or an autoimmune pattern can be present, several reported cases described a predominantly hepatocellular pattern [6,67,68,69,70,71]. On the other side, an autoimmune damage with autoantibodies (i.e., ANA, ASMA, and anti-LKM antibody), along with classic histologic characteristics of autoimmune hepatitis (i.e., interface hepatitis, lymphoplasmacytic infiltrate, and bridging fibrosis) has also been reported [72,73,74,75,76,77,78,79]. In particular, IFX immuno-mediated hepatotoxicity can resemble that of an AIH type I, with elevation of anti-nuclear (ANA), anti-smooth muscle (ASMA) and anti-double-strand DNA antibodies (anti-ds-DNA) [67]. Adar et al. in 2010 described the first case of ADA-induced AIH in a 36-year-old woman treated with ADA and leflunomide for PsA [76]. After discontinuation of both drugs, liver enzymes returned to normal values and, interestingly, hepatitis did not recur after ADA rechallenge [76]. More recently, 34 cases of DILI associated to TNF-α antagonists included in the United States DILI Network database (between 2003 and 2011) have been analysed [7]. The anti-TNF-α agent was the definite cause for only 1 case of DILI, whereas it was a probable cause and a very likely cause in 12 and 21 cases, respectively [7]. Again, 22 of 33 patients who underwent serologic analysis were positive for ANA and/or ASMA, of these 15 had histological features of AIH at liver biopsy [7]. The most common presentation of liver injury with an “autoimmune” phenotype was a higher peak level of alanine aminotransferase (ALT) [7]. The prognosis was usually favourable after drug suspension, even if in 12 cases corticosteroids were used [7]. Borman et al. described other two cases of severe AIH associated with anti-TNF-α therapy in RA patients. In both cases, the patients responded to the withdrawal of anti-TNF-α therapy and immunosuppressive treatment [80]. 11 cases of liver injury were described also in Iceland. In 8/11 of the cases, the causality between liver damage and anti-TNF-α was highly probable [81]. In the same country, a prospective study in the general population identified 96 patients with DILI from 2010 through 2011. Of note, the highest risk of liver injury was due to azathioprine and IFX, even if the percentage of cases due to these drugs among patients with DILI was small (4%) [82,83].

**Figure 2:**
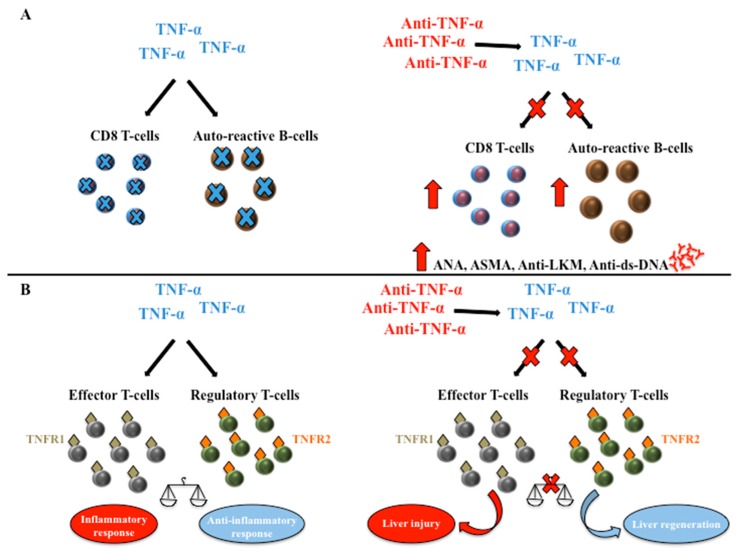
Working hypothesis for the anti-TNF-α-mediated mechanisms of liver damage. (**A**) TNF-α blockade could impair the normal suppression of auto-reactive B cell production and apoptosis of CD8 T cells leading to an increased lymphocyte presence. This could trigger the development of autoantibodies; (**B**) TNF-α can mediate a dual and opposing effect by acting on two TNF receptors (TNFR1 and TNFR2) expressed on T cells. TNF-α is able to stimulate effector T cell, mainly through TNFR1, which drives inflammatory response. On the other side, the activation of TNFR2 expressed on regulatory T cells leads to the prevention of autoimmunity and attenuation of inflammation. Thus, the blockade of TNF-α may either be able to determine further liver injury or regeneration by modulating the balance between effector and regulatory T cells. The direction of this response could also be influenced by the genetics and the immunological status of the host (e.g., cytokines, TNFR expression profile, cellular source of TNF). TNF-α, Tumor necrosis factor-α; TNFR1, Tumor necrosis factor-α receptor 1; TNFR2, Tumor necrosis factor-α receptor 2; ANA, anti-nuclear antibodies; ASMA, anti-smooth muscle antibodies; Anti-LKM, anti-liver kidney microsomal type 1 antibodies; Anti-ds-DNA, anti-double-strand DNA antibodies.

Despite the pathogenesis of AIH induced by IFX still being unclear, it has been suggested that TNF-α blockade could impair the normal suppression of auto-reactive B cell production and apoptosis of CD8 T cells leading to an increased lymphocyte presence. This could trigger the development of autoantibodies (i.e., ANA and anti-ds-DNA) [73,84]. Other studies hypothesized an immune dysfunction and liver repair process alteration caused by the immunoregulatory properties of TNF-α [68,85]. Indeed, TNF-α can mediate a dual and opposing effect by acting on two TNF receptors (TNFR1 and TNFR2) expressed on T cells, and promoting both hepatocyte necrosis in response to toxins and conversely increasing liver cell proliferation in appropriate conditions [86]. Specifically, TNF-α is able to stimulate effector T cells, mainly through TNFR1, which drives inflammatory response. On the other side, the activation of TNFR2 expressed on regulatory T cells leads to their expansion and consequently to the prevention of autoimmunity and attenuation of inflammation [87]. Thus, the blockade of TNF-α may either be able to determine further liver injury or regeneration by modulating the balance between effector and regulatory T cells. The direction of this response could also be influenced by the genetics and the immunological status of the host (e.g., cytokines, TNFR expression profile, cellular source of TNF) (Figure 2).

Cholestatic hepatitis induced by anti-TNF-α agents has also been described [6,69,88]. In one report, hepatic necrosis led to fulminant liver failure and urgent liver transplantation was needed [89]. Moreover, a case of hepatocellular carcinoma in a patient treated with anti-TNF-α in combination with azathioprine was reported [90]. Notably, some authors have observed the absence of hepatic cross-toxicity between IFX and the other anti-TNF-α agents [78,79]. This is likely due to the difference in their molecular structure. In particular, patients after an acute toxic hepatitis on IFX treatment and its discontinuation were treated with ETA or ADA for more than 2 years with no relapse of hepatitis [91,92]. Therefore, hepatotoxicity may not be a class effect of anti-TNF-α treatment but probably could be related to the development of antibodies against IFX. In the IBD setting, only few cases of liver toxicity related to IFX were published [74]. Menghini et al. described a case of a 44-year-old woman with CD who developed jaundice with a cholestatic liver disease after a single infusion of IFX [88], whereas more recently Ierardi et al. reported the case of a steroid-refractory UC patient who developed cholestatic acute liver damage (also in this case after a single infusion of IFX) resolved spontaneously within six weeks [69]. In both patients, antinuclear antibodies, alcohol intake, concomitant use of hepatotoxic drugs and all known viral and metabolic causes of hepatic injury were negative, likewise no serological and morphological findings of primary sclerosing cholangitis were observed. In a retrospective case series study, two patients were treated with IFX, whereas another patient was treated with ADA for IBD. All three patients had negative viral markers, normal autoimmune serologies, and normal biliary imaging studies. All patients showed a hepatocellular injury both biochemically and histologically, with negative autoantibodies. At liver biopsies, microscopic characteristics of hepatitis, without interface hepatitis or lymphoplasmacytic infiltration were observed. Liver inflammation normalized after IFX discontinuation and no serious injuries were reported. Notably, a successful transition from IFX to ADA was performed in one patient without relapse of either IBD or liver injury [93]. A retrospective cohort of 102 cases of elevated serum ALT in patients with IBD on anti-TNF-α therapy was also reported; 34 continued the anti-TNF-α medication, 14 stopped therapy and 4 started steroids. The 85% normalized their ALT after a median of 17 weeks including 28 (82%) of those who did not stop anti-TNF-α therapy. 10 patients were shifted to a second anti-TNF-α without recurrence. Overall, 48 cases were considered due to anti-TNF-α medications. The majority displayed autoimmune characteristics with positive serological markers [94].

Among all these reports, there are some clear differences, including the definition of hepatic injury and the patient populations studied. Conversely, there are also some notable similarities. First, IFX appears the most common cause, with ETA and ADA involved to much lesser degrees [95]. However, it should be taken into account that these three medications have been used for longer periods when compared with the newer drugs in this class and, thus, longer post-marketing surveillance intervals are available. Second, while multiple anti-TNF-α drugs have been associated with liver damage by sharing similar adverse effect features, it is clear that there is more than only a class effect involved. Indeed, some patients received anti-TNF-α without adverse events after reporting a liver injury caused by a different agent in the class [95]. Of note, one report described the case of a patient who developed an AIH from IFX and, after recovery, was treated again with IFX without developing a recurrence of liver injury [96].

**Figure 3:**
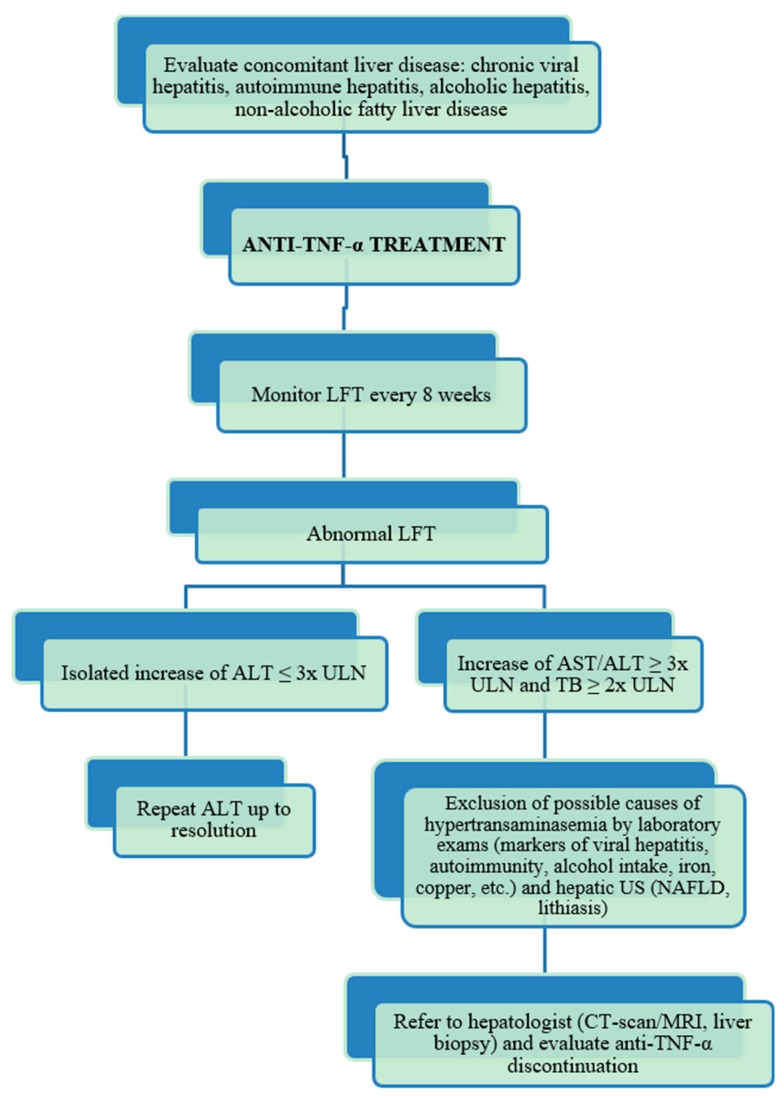
Algorithm for the diagnosis and management of anti-TNF-α-induced liver injury. TNF-α, tumor necrosis factor-α; LFT, liver function tests; ALT, alanine aminotransferase; ULN, upper limit of normal; US, ultrasound; NAFLD, non-alcoholic fatty liver disease; CT-scan, computed tomography-scan; MRI, magnetic resonance imaging.

In conclusion, these cases may alert physicians to the possibility of liver injury associated with the use of TNF-α blockers in an autoimmune setting, especially in the presence of pre-existent serological signs of autoimmunity such as ANA. While the incidence of liver injury due to anti-TNF-α therapy seems to be relatively low, the hepatic damage is nonetheless significant. Thus, in patients with an important elevation of transaminases or clinical signs consistent with acute hepatitis, anti-TNF-α should be stopped before the development of severe irreversible injury. In addition, based on current data, the mechanisms of liver toxicity may be different between IFX and ETA, according to their different structure. Moreover, ETA may be safe in patients who have presented liver toxicity while receiving IFX. In Figure 3 we suggest a clinical algorithm to follow for the cases of suspected anti-TNF-α induced hepatotoxicity.

## 6. Potential Beneficial Effects of Anti-TNF-α on Liver Diseases

### 6.1. Alcoholic Hepatitis (AH)

AH is a necro-inflammatory liver disease, observed in approximately 20% of heavy drinkers. Severe AH is associated with high morbidity and mortality rates [97,98]. In the pathogenesis of AH, several proinflammatory cytokines have been involved [99,100,101]. Among these, TNF-α has emerged as a key factor in the inflammatory process [101]. Data obtained from animal experiments have demonstrated that TNF-α exerts vascular effects by increasing vascular permeability and causing vasodilation [102]. Moreover, in animal models, antibodies to TNF-α attenuated liver injury and, mice lacking TNF-α receptor 1 did not develop alcohol-induced liver injury [103,104]. Also human data support a crucial role for TNF-α in the pathogenesis of AH. Indeed, circulating levels of TNF-α have been reported to be elevated in AH [105,106]. Moreover, plasma TNF-α levels were significantly higher in AH patients who subsequently died than those who survived [106]. The therapy for severe AH has three objectives: the treatment of alcoholism, nutritional support, and specific pharmacological treatment [97,107,108]. Actually, the most important endpoint is to reduce early mortality. Several studies showed that corticosteroids are the standard treatment for severe AH, since they were able to reduce the 1- and 2-month mortality [109,110]. Approximately 75% of patients with DF ≥ 32 were responders to this therapy [110]. Prednisolone, 40 mg/day for 28 days (with or without a 2-week taping), is the most widely recommended pharmacological regimen to treat AH [111]. Recently, a meta-analysis including 418 patients with severe AH compared 28-day survival rate between corticosteroid- and non-corticosteroid-treated patients. Authors, analysing the response to treatment by the Lille model (a specific prognostic model that combines six reproducible variables: age, renal insufficiency, albumin, prothrombin time, bilirubin, and evolution of bilirubin at day 7), showed that corticosteroids had a significant effect on 28-day survival rate mainly in complete and partial subgroup responders (Lille score ≤ 0.16 and 0.16–0.56, respectively), but not in null responders (Lille score ≥ 0.56) [112]. Nevertheless, the decreasing effect of TNF-α and IL-8 due to glucocorticoids is delayed, and this might contribute to the high short-term mortality in these patients (~25%) [113]. Moreover, 40% of these patients died in the 6 months following the AH onset [112,114]. Finally, it is widely known that corticosteroids have several side-effects and should be used with caution especially in immunocompromised patients with high risk of gastrointestinal bleeding. In this scenario, the use of TNF-α blockers was considered an attractive approach for AH treatment. The efficacy of the combination of steroids with IFX has been investigated in two pilot randomized trials [115,116]. In the first study, Spahr et al. [115] enrolled 20 patients with biopsy-proven severe AH and treated them with prednisone 40 mg/day for 28 days and either infliximab 5 mg/kg intravenously (IV) (*n* = 10) or placebo (*n* = 10) at day 0. The authors excluded patients with DF > 55. At day 28, there was a significant improvement in the IFX group for both Maddrey’s score and IL-6 and IL-8 levels. Conversely, in the placebo group no significant difference was observed for these parameters compared with baseline. IFX was well tolerated and there were no significant differences between the two groups in terms of side effects [115]. In the second trial [116], 36 patients with severe AH were randomized to receive prednisolone 40 mg/day in combination with IFX 10 mg/kg at weeks 0, 2, and 4 (group A), or placebo (group B). The aim of this study was to evaluate the efficacy of the combination therapy in terms of 2-month mortality rate reduction. After randomization, there were seven deaths in the IFX group and three in the placebo group. The mortality at two months was higher in group A than in group B (39% vs. 18%). For this reason, the study was stopped, and authors concluded that three infusion of IFX 10 mg/kg in association with prednisolone may be harmful in these severe AH patients. Other studies have tested the efficacy and safety of a single dose of IFX alone for the treatment of severe AH [117,118]. In the first trial by Tilg et al. [117], 12 patients with biopsy-confirmed AH and a Maddrey’s DF > 32 were treated with a single infliximab infusion (5 mg/kg). Two patients died from infective complications within the first 4 weeks while among the remaining patients the median survival resulted in being 15 months [117]. Serum bilirubin levels, Maddrey’s score, neutrophil count and C-reactive protein (CRP) decreased significantly within the first month [117]. Interestingly, authors observed the stability of both bilirubin levels and DF during the 14 days after IFX infusion. These began to decrease only after 21 days, suggesting that anti-TNF-α biological effect is delayed compared to corticosteroids (observed within 7 days). On the other hand, IL-6 and IL-8 levels decreased immediately after IFX administration. Another study evaluated the effect of a single injection of IFX at a dosage of 5 mg/kg in 10 patients with severe AH [118]. IFX reduced significantly serum bilirubin, CRP, white cells count, IL-6 and IL-8 plasma levels [118]. Moreover, 24 h after IFX injection the mean hepatic venous pressure gradient (HVPG) decreased [118]. More recently, an open label trial has confirmed that a single dose of IFX may be associated with a clinical and biochemical improvement in patients with severe AH [119]. In this study 19 patients were treated with a single dose of IFX 5 mg/kg and then followed for two months. By the end of one month two patients died from renal failure leading to a 1-month survival of 89%. After 2 months, another four patients died (causes of death were: sepsis, disseminated tuberculosis, disseminated intravascular coagulation, encephalopathy) with a 2-month survival of 68%. Absence of hepatic encephalopathy at admission, Lille score and delta bilirubin at day 7 (DBD7) significantly predicted survival [119]. Finally, also ETA has been used in a randomized, double-blinded, placebo controlled trial [120]. The incidence of serious adverse events such as infections was significantly higher in the group treated with the anti-TNF-α agent compared to placebo (34% versus 9%, *p* = 0.04) as well as the 6-month mortality rate (58% versus 23%, respectively, *p* = 0.017) [120]. In conclusion, at present IFX and, in general, anti-TNF-α agents are not recommended for AH treatment, except for carefully designed clinical trials. However, some studies have demonstrated that a single IFX dose is associated with an improvement in severity and survival, whereas the combination of IFX with steroids is potentially more harmful. Anyway, safety (in particular infections) remains the first concern. Certainly, large randomized controlled studies are needed to determine whether anti-TNF-α agents may have a role in the treatment of AH.

### 6.2. Non-Alcoholic Fatty Liver Disease (NAFLD)

NAFLD includes a large spectrum of clinical and pathological liver conditions, including simple steatosis, non-alcoholic steatohepatitis (NASH) and fibrosis [121,122]. NAFLD is a prevalent health problem and has become the most common cause of abnormal liver function in several world regions, frequently leading to severe hepatic insufficiency and hepatocellular carcinoma [123,124,125]. The exact prevalence of NAFLD and NASH are not well known, but roughly is 25–30% and 2–3%, respectively [123,124]. In particular, NASH is characterized by diffused fatty infiltration, lobular inflammation and ballooning degeneration in the liver. The pathogenesis of NASH is not completely elucidated. It has been proposed that various cytokines play a crucial role in the process from steatosis to NASH. In this scenario, TNF-α has emerged as a key inducer of nutrient- and obesity-associated NASH [126,127]. Patients with NASH have generally significantly higher levels of serum TNF-α and IL-6 than those observed in patients with simple steatosis [128]. Tomita et al. [129] showed that TNF-α is able to induce the activation of stellate cells, the matrix gene expression, and the matrix remodelling, which are important events during the onset of NASH. Moreover, this cytokine could also promote insulin resistance by the activation of JNK and IκBK, two intracellular serine kinases that inhibit important substrates of the insulin signalling pathway [130]. As such, the strategies aiming at blocking the effect of TNF-α in the liver may be potentially useful for the treatment of NASH. In an experimental rat model of NASH induced by methionine and choline deficient diet, the treated group received a single intraperitoneal dose of infliximab (4 mg/kg per week) whereas the control group received intraperitoneal injections of sterile saline solution [131]. Anti-TNF-α decreased AST, ALT and TGF-β levels. Moreover, hepatic inflammation, necrosis and fibrosis decreased in IFX-treated animals compared with placebo (*p* < 0.05) [131]. The effects of IFX on liver steatosis, fibrosis, and insulin signal transduction were evaluated also in another group of rats fed with a high-fat diet [132]. Authors demonstrated that 10 days of therapy with IFX significantly decreased liver levels of TNF-α, IL-6, IL-1β, IL-10, and SOCS-3. Furthermore, a reduction of fat deposition and fibrosis, and an improvement of insulin signal transduction were observed [132]. There are currently no FDA-approved treatments for NASH. Anti-TNF-α agents have not yet been evaluated for this indication. However, one interesting case of rapid normalization of liver biochemical parameters in a patient with NASH during treatment with ADA for concomitant rheumatoid arthritis was described [133].

In conclusion, although no data in patients with NASH are yet available, several experimental data seem to confirm that the blockade of TNF-α might be an important target for the treatment of NASH. Certainly, we are looking forward to new studies designed to evaluate the role of anti-TNF-α agents for the treatment of NASH.

### 6.3. Autoimmune Hepatitis (AIH)

AIH is a relative rare chronic liver disease that affects mainly woman and is characterized by elevated transaminases, hypergammaglobulinaemia, circulating autoantibodies, interface hepatitis at liver histology. If untreated, it often leads to cirrhosis, liver failure and death [12]. In the absence of cirrhosis, standard treatment comprises azathioprine and prednisolone, and leads to complete biochemical response in up to 77% of patients after six months of treatment [134]. However, 5% of patients experience intolerance or toxicity and 10% do not sufficiently respond to standard treatment options [135]. Alternative treatments can include budesonide [136], mycophenolate mofetil (MMF) [137], 6-thioguanine [138], cyclophosphamide [139], cyclosporine A [140] or tacrolimus [141]. However, these treatments have mostly been described in small case series rather than controlled trials, and have demonstrated only variable effectiveness. Therefore, the identification of efficient rescue treatment options is urgently needed for these difficult-to-treat patients.

Infliximab (5 mg/kg at day 0, weeks 2 and 6 and, thereafter, every four to eight weeks depending on laboratory and clinical course) may be considered as rescue treatment in patients (especially pediatric) with difficult-to-treat AIH, even if therapy may be associated with infection complications [11,142].

In general, patients with autoimmune liver diseases display a different cytokine expression pathway compared with healthy patients, with increased serum levels of proinflammatory cytokines, such as IL-6, IL-8, and TNF-α [143]. Furthermore, a genetic polymorphism of TNF-α gene has been evidenced in type I AIH [144]. This polymorphism is linked to high levels of TNF-α and triggers a type 1 cytokine response. Interestingly, young patients with this polymorphism have been shown to experience a reduced response to corticosteroids. Moreover, increased TNF-α levels could be responsible for the necrotic process of hepatocytes. In this scenario, IFX is able to impair the pro-necrotic activity of activated lymphocytes. This could explain the observation of a better response to IFX in children with refractory AIH [65,144].

Weiler-Normann et al. [11] described the first series of 11 patients with difficult-to-treat AIH who received rescue treatment with IFX. In these patients, IFX showed good remission rates (in more than 60% of patients), comparable to those of patients with AIH that can be managed with standard therapy with a tolerable safety profile. Furthermore, Rajanayagam et al. reported the clinical case of a 10-year old girl with type I AIH refractory to azathioprine, mycophenolate mofetil, and tacrolimus, requiring a continuous corticosteroid therapy [142]. After 3 weeks from the beginning of IFX, a biochemical and clinical amelioration with steroid-sparing therapy were observed [142]. Of note, some authors underlined that strong immunosuppression is associated with severe infectious complications, especially in cirrhotic patients [12]. Patients with difficult-to-treat autoimmune hepatitis are generally at higher risk of infectious complications following intense immunosuppressive treatment, compared with those responding promptly to standard treatment. Moreover, a condition of liver cirrhosis increases the infection risk [145]. Interestingly, in the report of Weiler-Normann et al. [11], only 2/11 patients required hospitalization and complete recovery was achieved under standard treatment.

Anyway, no strong recommendation can be drawn from these small sample size studies. On the other side, anti-TNF-α antibodies may also induce an immune-mediated liver disease resembling AIH, as previously discussed.

### 6.4. Primary Biliary Cholangitis (PBC)

PBC is a chronic progressive liver disease characterized by serological presence of the antimitochondrial antibodies (AMA) with a title of 1/40 or greater, chronic non-suppurative destruction with progressive loss of intrahepatic small bile ducts on liver histology, resulting in progressive fibrosis and cirrhosis. PBC is considered an immunologically mediated disease where the TNF-α plays a prominent pathogenic role [10]. The unique data published on the use of IFX in PBC are collected in patients with RA and PBC. While TNF-α inhibitors are a well-established treatment option for RA, the experience of using such regimens when inflammatory arthritis coexists with PBC is inconclusive. Garyfallos et al. [10] reported a case of a female patient with RA and concomitant PBC with poor clinical response to conventional treatment. IFX use led to a significant clinical improvement of RA and to the stabilization of liver function. Spadaro et al. [146] reported a poor clinical response for arthritis and persistence of liver function test abnormalities during IFX therapy, which returned to normality after the switch to ETA. Similarly, ETA led to adequate control of RA and PBC in another published case [147]. Del Ross et al. reported the case of an old female affected with PsA and an overlapping PBC and PSC treated with ADA for 28 months. This treatment led to the clinical amelioration in arthritis symptoms and in nail lesions as well as lowered cholestasis indices, overall improving the symptoms of both cholangiopathies [148].

Furthermore, reversible cholestatic liver disease has been noticed in a few patients receiving IFX for various autoimmune disorders [88].

These reports could suggest a common pathogenic pathway for these diseases. In this scenario, the use of anti-TNF-α could be useful in some subsets of autoimmune colangiopathies. In particular, among these groups, those with a concomitant inflammatory arthropathy could have a successful response to anti-TNF-α drugs.

## 7. Conclusions

Anti TNF-α agents represents a paramount treatment for several chronic inflammatory diseases. Although their clinical use and safety risk for hepatic diseases has not been completely explored, they have undoubtedly a role on liver pathophysiology. Indeed, they possess a dichotomous impact with both therapeutic and toxic effect depending on the immunological status and the concomitant diseases of the host. It should be underlined that no randomized controlled trials or well-conducted observational studies exist that assess, for example, the safety of TNF-α blockers in Hepatitis B or Hepatitis C or their overall hepatotoxic potential (even large case series from established networks do not provide enough information to quantify the risk of hepatotoxicity). Future studies should also better define the mechanisms of liver injury linked to anti-TNF-α drugs. More importantly, reliable factors are crucially needed to predict the risk of adverse hepatic effects. Conversely, the investigation on specific host variables that are connected to TNF-α involvement in inflammatory liver diseases could open new therapeutical horizon to the selection of patients who would benefit from anti-TNF-α use.

## Figures and Tables

**Table 1:** Anti-tumor necrosis factor (anti-TNF-α) and the liver.

Harmful Effects of Anti-TNF-α Agents	Potential Beneficial Effects of Anti TNF-α Agents
Reactivation of HBV infection ✓ HBsAg carrier ✓ Occult carrier (anti-HBc+)	Alcoholic hepatitis (AH)
Hepatotoxicity ✓ Direct liver injury (idiosyncratic injury) ✓ Immuno-mediated liver injury (autoimmune hepatitis)	Non-alcoholic fatty liver disease (NAFLD)
	Autoimmune hepatitis (AIH)
	Primary biliary cholangitis (PBC)

## Abbreviations

IBD	Inflammatory Bowel Diseases
CD	Crohn’s Disease
UC	Ulcerative Colitis
TNF-α	Tumor Necrosis Factor
RA	Rheumatoid Arthritis
JIA	Juvenile Idiopathic Arthritis
AS	Ankylosing Spondylitis
PsA	Psoriatic Arthritis
IFX	Infliximab
ADA	Adalimumab
CER	Certolizumab Pegol
ETA	Etanercept
GOL	Golimumab
Ig	Immunoglobulin
HBV	Hepatitis B Virus
HCV	Hepatitis C Virus
AH	Alcoholic Hepatitis
NAFLD	Non-Alcoholic Fatty Liver Disease
AIH	Autoimmune Hepatitis
PBC	Primary Biliary Cholangitis
ECCO	European Crohn’s and Colitis Organization
ALT	Alanine Aminotransferase
IFN	Interferon
LT	Liver Transplantation
FDA	Food and Drug Administration
MMF	Mycophenolate Mofetil
DILI	Drug Induced Liver Injury
CRP	C-reactive protein
NASH	Non-Alcoholic Steatohepatitis
TNFR	TNF Receptor
